# A Rare Etiology of Esophageal Obstruction: Esophageal Clot Formation After Teeth Extraction

**DOI:** 10.7759/cureus.37892

**Published:** 2023-04-20

**Authors:** Pradeep R Kathi, Brendan Andres

**Affiliations:** 1 Internal Medicine and Gastroenterology, University of Arizona, Tucson, USA; 2 Internal Medicine, University of Arizona College of Medicine, Tucson, USA

**Keywords:** esophageal obstruction, oral hemorrhage, dental extraction, balloon dilation, esophageal stricture

## Abstract

Esophageal obstruction represents an endoscopic emergency owing to the high mortality rate associated with complications, including perforation and airway compromise. While typically caused by food or foreign body ingestion, esophageal clot represents a rare etiology of obstruction. We present a case of esophageal obstruction in the context of an anastomotic stricture and chronic anticoagulation for atrial fibrillation caused by clot formation due to oral hemorrhage after dental extractions. Clot retrieval was accomplished via endoscopic suction, and balloon dilation of the anastomotic stricture was performed to prevent recurrence. Our case illustrates the importance of considering oral hemorrhage, therapeutic anticoagulation, and esophageal strictures as risk factors for esophageal obstruction due to clot formation in order to make a timely diagnosis and treatment of this potential endoscopic emergency.

## Introduction

Foreign body ingestion and food bolus impaction are the most common etiologies of esophageal obstruction. While previously reported in the context of reflux esophagitis and Mallory-Weiss tear, obstructive esophageal clot formation is rare [1,2]. Patients can present with dysphagia, odynophagia, sialorrhea, and intolerance of liquids. Complete esophageal obstruction is an endoscopic emergency because without the ability to clear esophageal contents, patients may aspirate leading to pneumonia and airway compromise. Pressure injury and ischemia due to impaction can additionally cause esophageal perforation. The mortality rate of uncomplicated esophageal impaction is 0.85%; however, it approaches 20% when combining all complications and 40% with perforation [3-5].

Furthermore, esophageal strictures can potentiate esophageal obstruction due to reduced esophageal diameter and impaired peristalsis. Anastomotic strictures specifically occur due to postoperative scarring and altered blood supply. Patients who have undergone esophagectomy for esophageal adenocarcinoma, especially those who have undergone the transhiatal approach, are at increased risk for anastomotic stricture formation [6,7]. We describe the unique case of a patient with a history of anastomotic esophageal strictures who developed esophageal obstruction due to occlusive clot formation caused by oral hemorrhage after dental extractions.

## Case presentation

A 78-year-old male with a history of transhiatal gastroesophagectomy with gastric pull-up for adenocarcinoma of the gastroesophageal junction complicated by anastomotic stricture requiring endoscopic dilation, atrial fibrillation on apixaban 5 mg twice daily, and chronic kidney disease stage 3 was sent to the emergency department from oral surgery clinic due to inability to tolerate any oral intake and oral bleeding from the sites of teeth extraction. One day before his presentation, the patient had several teeth extracted, and he had ongoing oozing from the sites of teeth extraction since the procedure. He ingested a single pill of acetaminophen soon after the teeth extraction for pain. However, the patient soon experienced dysphagia and odynophagia to a clear liquid diet. He denied having solid food intake. Apixaban had been held for two days prior to the oral surgery, and the patient had not restarted the medication prior to his presentation to the emergency department.

**Figure 1:**
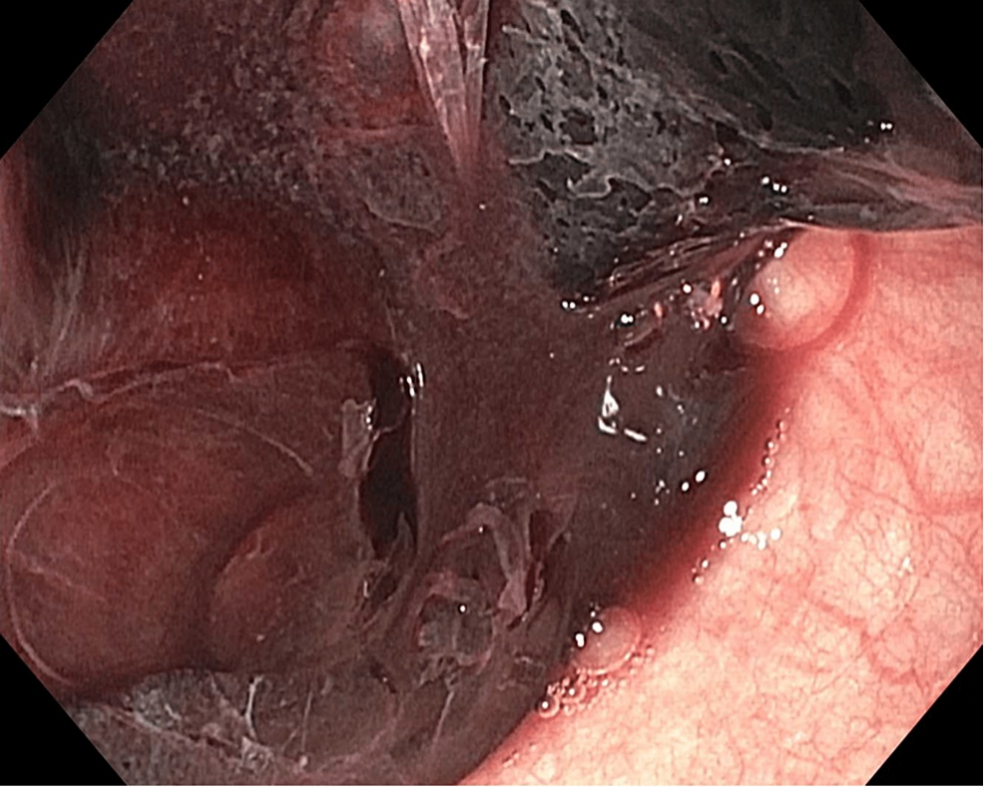
EGD depicting esophageal clot proximal to anastomotic stricture EGD, esophagogastroduodenoscopy

**Figure 2:**
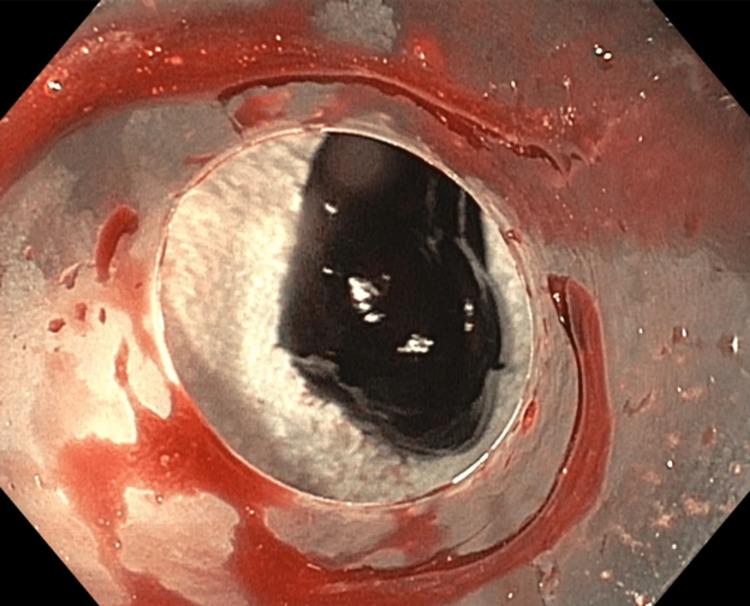
Esophageal clot after removal via endoscopic suction

**Figure 3:**
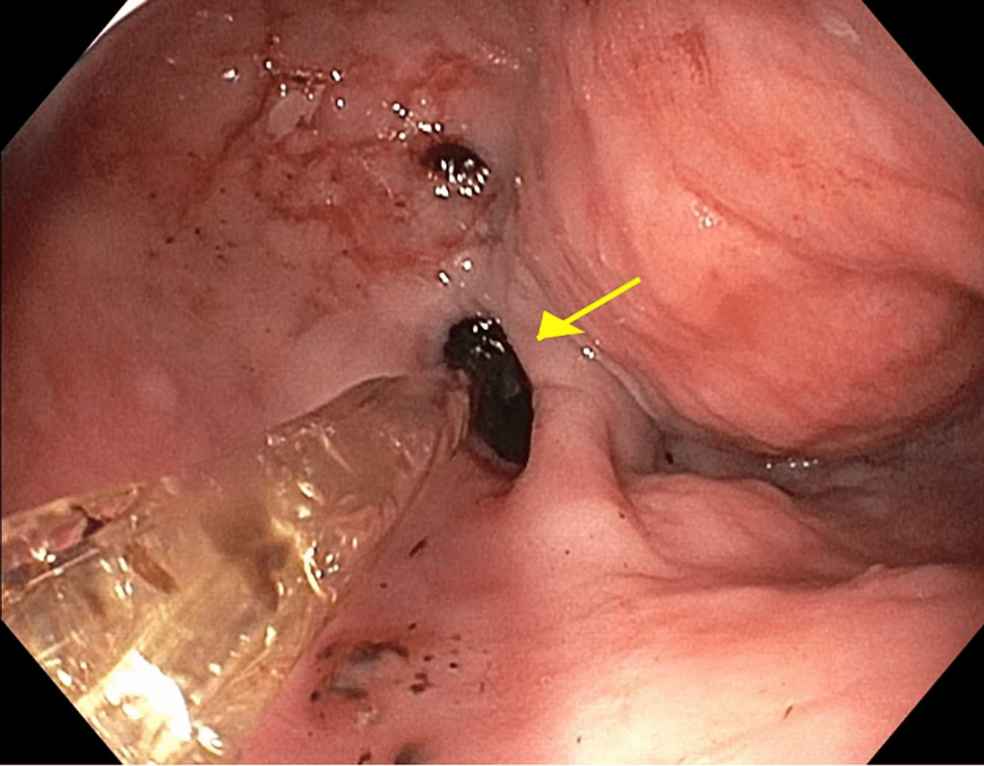
EGD depicting anastomotic stricture (yellow arrow) after proximal clot removal and insertion of balloon dilator EGD, esophagogastroduodenoscopy

Due to the patient’s inability to swallow the oral secretions, an emergent esophagogastroduodenoscopy (EGD) was performed. The EGD revealed a large amount of blood clots at the site of the anastomotic stricture, which was located at 20 cm from incisors without active esophageal hemorrhage (Figure 1). Retrieval of the clots was not successful with a Roth net, and the suction cap was used to remove the clots instead (Figure 2). The anastomotic stricture measured approximately 10 mm in diameter (Figure 3), and it was dilated to 15.5 mm with a through-the-scope dilator. There was no evidence of active or prior esophageal hemorrhage. The patient was able to resume a regular diet after the EGD.

## Discussion

This case highlights a unique combination of risk factors that contributed to luminal obstruction of the esophagus. To our knowledge, this is the first reported case of complete esophageal obstruction due to clot formation caused by an oral hemorrhage. Other contributing factors included the patient’s history of anastomotic strictures after esophagectomy and the use of oral anticoagulants. Nevertheless, the obstruction was amenable to standard endoscopic techniques including suction cap extraction and balloon dilation.

Esophageal strictures increase the risk of obstruction due to food, foreign body, and rarely, hemorrhage [6,7]. Though an uncommon cause of benign esophageal stricture, anastomotic strictures have been observed in 9-48% of patients who have undergone esophagectomy, and recurrence rates approach 50% [8]. Hemorrhage is further potentiated by oral anticoagulants prescribed for stroke prophylaxis for atrial fibrillation. However, apixaban has been associated with lessened frequencies of nonmajor and major hemorrhage [9]. With a half-life of eight to 12 hours, apixaban may be held for 24 hours prior to procedures with low bleeding risk and resumed after 24 hours in patients with stage 3 or less chronic kidney disease [10,11]. While the specific reason for the patient’s prolonged oral hemorrhage remains unclear as the apixaban was appropriately dosed and held prior to the procedure, the patient likely experienced an asymptomatic recurrence of his anastomotic stricture that allowed for clot accumulation and subsequent complete esophageal obstruction.

Intolerance of secretions and liquids is a clinical sign of complete esophageal obstruction and necessitates emergent endoscopy within two to six hours [12,13]. Prompt diagnosis and intervention is key because adverse patient outcomes including rates of complications and duration of hospitalization increase after 24 hours [14]. Since the patient presented with intolerance of liquids, emergent endoscopy was performed due to concerns of complete esophageal obstruction. Upon discovery of the obstructive clots, removal was accomplished with simple extraction via a suction cap due to provider preference. Evidence suggests that cap-assisted endoscopic removal of esophageal foreign body has been associated with better technical success and en bloc removal and a shorter procedure time compared to conventional methods [15]. Other available methods of endoscopic foreign body extraction include rat-tooth and alligator forceps, polypectomy snares, polyp graspers, Dormier baskets, and retrieval nets [12]. Standard balloon dilation was then performed to dilate the stricture to approximately 15 mm for relief of dysphagia and to decrease the risk of recurrent obstruction [16].

While more commonly caused by food and foreign bodies, this case emphasizes that the clinician should consider clot formation as an etiology of esophageal obstruction in the appropriate clinical setting. Urgent or emergent endoscopy should then be pursued depending on evidence of partial or complete esophageal obstruction, respectively. Esophageal obstructions can be treated via endoscopic extraction using a variety of techniques and devices. If esophageal strictures are present, then dilation should be attempted to reduce the risk of repeated obstruction.

## Conclusions

Though uncommon, clot formation that completely obstructs the esophageal lumen is an endoscopic emergency. The clinician should maintain awareness of risk factors for hemorrhage and esophageal narrowing, which may contribute to obstructive clot formation. Specific risk factors may include oral anticoagulants, dental procedures, and a history of esophageal strictures. Once observed, clots may be removed endoscopically via standard suction techniques including suction cap extraction, and precipitating strictures should be dilated to prevent recurrence.
